# Resilient distributed model-free adaptive traffic signal control via controller dynamic linearization under DoS attacks

**DOI:** 10.1371/journal.pone.0342069

**Published:** 2026-02-12

**Authors:** Ye Ren, Haoyang Sun, Ting Lei, Honghai Ji, Shida Liu, Li Wang

**Affiliations:** 1 School of Electrical and Control Engineering, North China University of Technology, Beijing, China; 2 School of Electrical and Information Engineering, Zhengzhou University of Light Industry, Zhengzhou, China; Southwest Jiaotong University, CHINA

## Abstract

The increasing complexity and persistent network security challenges in traffic signal control are key issues requiring urgent attention to meet growing traffic demands. To address these issues, this paper proposes a resilient distributed model-free adaptive traffic signal control strategy (CDL-DMFAC) that integrates controller dynamic linearization (CDL) with multi-agent modeling. In the proposed framework, each signal phase at an intersection is modeled as an independent agent, and a compact form dynamic linearization (CFDL) is employed to construct an unknown ideal controller, enabling balanced control of multi-phase queue lengths. Furthermore, a denial-of-service (DoS) attack compensation mechanism is designed to mitigate the negative impact of communication interruptions or delays on signal timing decisions. Experimental results show that CDL-DMFAC effectively suppresses queue growth and delay accumulation under various attack intensities, with its performance advantage becoming more pronounced as attack severity increases. Notably, under the most challenging scenario—high traffic demand with multiple intersections simultaneously subjected to DoS attacks—the proposed method achieves reductions of 28.3% in average queue length and 36.32% in average waiting time compared to conventional signal control methods. These results highlight the method’s strong resilience against attacks, operational stability, and potential for deployment in larger-scale urban traffic networks.

## Introduction

As the “lifeline” that sustains the normal operation of cities, urban transportation systems are confronting dual pressures from accelerated urbanization and imbalanced allocation of transportation resources. The contradiction between limited road resources and continuously growing traffic demand has become increasingly prominent. Against this backdrop, traffic signal control, as a critical technical measure for alleviating traffic congestion and improving road capacity efficiency, has long attracted extensive attention from both the academic and engineering communities.

To date, various urban traffic signal control strategies have been developed, including fixed-time control [1], SCOOT [2], SCATS [3], proportional-integral-derivative (PID) control [4], queue length feedback (QLFB) control [5], model predictive control (MPC) [6], and reinforcement learning (RL) approaches [7,8]. Although these centralized control strategies have achieved remarkable results in both theoretical research and practical applications, the continuous growth in the scale and complexity of traffic systems has gradually revealed several limitations, such as high computational resource consumption, increased difficulty in achieving global optimization, and challenges in maintaining long-term system stability. In contrast, distributed control methods, with their architectural flexibility and stronger operational robustness, have demonstrated unique advantages in the real-time regulation of large-scale traffic networks, offering a promising technical pathway and development direction for urban traffic signal control.

In recent years, multi-agent-based control methods have been extensively studied and applied in the field of traffic signal control. The multi-agent systems (MASs) can effectively capture the interactions and cooperation among different traffic participants, thereby enabling distributed optimization of traffic signals. This approach offers strong flexibility and high scalability, making it particularly suitable for dynamic and complex urban traffic environments. In related studies, different scholars have emphasized various aspects. For example, [9] proposed a distributed stochastic model predictive control method that determines the optimal stochastic signal timing through a decentralized strategy; [10] introduced mathematical programming into intersection signal timing optimization, effectively preventing network oversaturation and reducing delays; [11] developed a distributed adaptive cooperative control method and applied Lyapunov analysis to shorten queue lengths; [12] coupled MASs with a cellular automata model to achieve the integration of microscopic simulation and macroscopic regulation; [13] introduced a value decomposition-based spatio-temporal graph attention multi-agent deep reinforcement learning approach, which performed well in heterogeneous road networks; [14] established a knowledge-sharing protocol among agents to enhance collaborative signal optimization capabilities; [15] integrated Nash equilibrium with reinforcement learning, achieving significant reductions in waiting time; and [16] applied multi-agent deep reinforcement learning to environmentally friendly traffic control, aiming to minimize greenhouse gas emissions. Similarly, deep reinforcement learning has also been effectively utilized for multi-objective optimization in other complex network systems, such as micro-grid energy dispatch [17]. Furthermore, advanced resilient control strategies—such as fault-tolerant cooperative control and safety control with guaranteed performance—have been systematically established in other multi-agent domains like unmanned aerial vehicles (UAVs) [18,19]. These foundational works provide valuable methodological insights for enhancing the reliability of distributed control systems against disturbances and faults.

However, these multi-agent-based traffic signal control methods still have certain limitations. Model-based multi-agent approaches [9–12] face difficulties in constructing accurate models, making it challenging to strike a balance between model accuracy and computational complexity, and they exhibit insufficient adaptability to rapidly changing traffic environments. Although reinforcement learning-based multi-agent methods [13–16] show potential in terms of adaptability, they heavily rely on large amounts of high-quality data, require long training times, possess complex structures with limited interpretability, and have constrained adaptability to extreme traffic conditions and rare events.

To overcome the aforementioned limitations, this study introduces the model-free adaptive control (MFAC) method. The primary strength of MFAC is that it does not require an accurate system model; instead, it achieves control by dynamically learning the input–output relationship, which effectively mitigates model mismatch issues. Considering the strong nonlinearity and time-varying characteristics of traffic systems, MFAC employs pseudo partial derivative (PPD) to construct an equivalent dynamic linearization (DL) model, enabling online estimation of control parameters within a closed-loop system. By iteratively optimizing these parameters using real-time data, MFAC improves sample efficiency. Benefiting from these features, MFAC has been successfully applied in various domains, including nonlinear multi-agent systems, rail transit, autonomous driving, power systems, medical devices, and motor drives [20–27], demonstrating its broad adaptability to complex dynamic systems.

In the field of traffic control, MFAC has achieved notable results. For instance, [28] proposed a model-free adaptive iterative learning scheme for dynamically optimizing macroscopic traffic flow control parameters; [29] combined active disturbance rejection control with MFAC to achieve bus trajectory tracking; [30] introduced a hierarchical peripheral control strategy to reduce computational complexity; and [31] developed a predictive control method based on distributed model-free adaptive control (DMFAC) to address model mismatches in multi-area road networks; [32] proposed a model-free adaptive iterative learning boundary control scheme, which demonstrates stronger robustness and fault tolerance in multi-area traffic networks.

However, the aforementioned MFAC-related studies largely rely on controller structures based on DL and require a pre-defined cost function to determine the control law, which not only increases design complexity but also reduces the flexibility of the control strategy. To simplify the design process, some researchers have proposed the controller dynamic linearization (CDL) method [33–35], developed under the assumption of an ideal controller. The method assumes the existence of an ideal controller and integrates the cost function into the estimation of control gain parameters, thus enabling control strategy adjustments without the need for an explicitly designed cost function, while achieving more accurate target tracking. This feature is particularly important in traffic signal control scenarios that require real-time updates of control strategies.

As urban traffic signal control systems become increasingly networked and interconnected, security vulnerabilities have emerged as a critical concern. Among various cyber-attacks, denial-of-service (DoS) attacks are particularly destructive, potentially disrupting signal timing (e.g., shortening green light durations or indefinitely extending red lights), causing congestion, reducing traffic efficiency, and increasing safety risks. In severe cases, they can lead to the complete paralysis of the entire traffic control system. The challenge of ensuring resilience against cyber-attacks is not unique to traffic systems; for instance, advanced strategies like homomorphic encryption have been developed to secure distributed energy management in micro-grids under similar threats [36].

While several studies have explored the performance of MFAC under DoS attacks, such as [37] using event-triggered MFAC for nonlinear systems with sensor faults and DoS, and [38] proposing improved dynamic linearization and attack compensation for DMFAC, these works mainly address industrial and generic distributed control systems. They have not specifically incorporated DoS defense mechanisms into the context of traffic signal control, which involves real-time adjustments and multi-intersection coordination. Thus, integrating robust DoS defenses into traffic signal control systems remains an area that requires further research and development.

Based on the above analysis, this paper proposes a resilient distributed CDL-DMFAC traffic signal control method for networked traffic signal control systems under DoS attacks. The main contributions of this work are summarized as follows:

1. **Distributed data-driven modeling:** Each signal phase of an intersection is regarded as an independent agent, and a distributed input–output relationship equivalence is established via dynamic linearization.
2. **Compact form dynamic linearization (CFDL) controller design:** The CFDL technique is employed to construct an unknown ideal learning controller, thereby achieving the objective of balanced control of multi-phase queue lengths.
3. **DoS attack compensation mechanism:** A compensation mechanism for communication interruptions and delays is designed to effectively mitigate the negative impact of DoS attacks on signal timing, thereby enhancing the stability and resilience of the system under complex network environments.

## Problem formulation

### Topology graph description of MASs

When modeling the directional agents at a single signal-controlled intersection, this study first introduces the foundational graph theory concepts, which will not be elaborated further hereinafter. For the network structure of the multi-agent system, the following key definitions apply: *N* agent nodes are interconnected through a directed graph G={V,E,A}. Here, V={1,2,…,N} represents the set of agents; E=V×V denotes the edge set formed by communication channels between agents, where ’×’ signifies the Cartesian product. An edge (j,i)∈E indicates an open information channel from agent *j* to agent *i*. The adjacency matrix $A=[a_{ij}]\in {\mathbb{R}}^{N\times N}$ encodes the adjacency weights from agent *j* to agent *i*. Specifically, *a*_*ij*_ = 1 indicates the existence of a connection from agent *j* to agent *i*, while *a*_*ij*_ = 0 implies no connection. The set $N_{i}=\{j\in V|(j,i)\in E\}$ denotes the neighbor set of agent *i*, which comprises all agents *j* that have an outbound communication link directed toward agent *i*. This set represents the agents from which agent *i* can directly receive information. The in-degree of the *i*-th agent is defined as $d_{i}=\sum_{j=1}^{N}a_{ij}$. The in-degree matrix $D=\text{diag}\{d_{i}\}\in {\mathbb{R}}^{N\times N}$, and the Laplacian matrix of the directed graph *G* is given by *L* = *D*−*A*.

### Cooperative control objective

**Fig 1:**
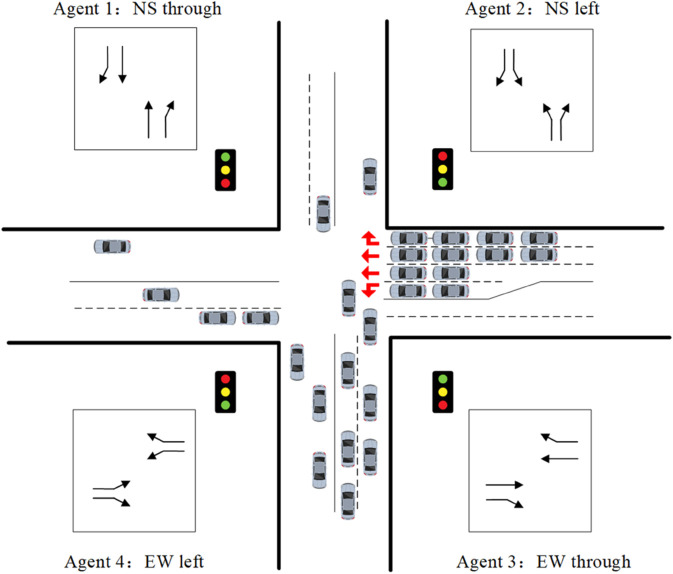
Phase division of an isolated signalized intersection.

For an isolated signalized intersection, its traffic operations can be categorized into four fundamental phases: north-south through, north-south left-turn, east-west through, and east-west left-turn, as illustrated in Fig 1. To capture the operational state of the intersection, a nonlinear discrete-time queue length model for each phase is formulated as follows:

$$l_{i}\left(k+1\right)=I_{i}\left(l_{i}\left(k\right),g_{i}\left(k\right)\right)$$ (1)

Among them, *l*_*i*_(*k*) denotes the queue length of the *i*-th phase at the intersection in the *k*-th cycle, *g*_*i*_(*k*) denotes the green time of the *i*-th phase at the intersection in the *k*-th cycle, $I_{i}(\cdot )$ is an unknown nonlinear function.

Remark 1: To ensure traffic safety and prevent conflicts, the four phases illustrated in Fig 1 are executed sequentially in a fixed cycle structure. This sequential execution inherently enforces a mutual exclusion logic: when a specific phase *i* (e.g., North-South Straight) is active (green), all other conflicting phases are strictly maintained at red. Consequently, the proposed controller optimizes the green time duration *g*_*i*_(*k*) for each phase without altering this underlying safety interlock logic, ensuring that conflicting traffic flows are never released simultaneously.

Within the coordinated control framework, the four phases are treated as four agents, forming a directed graph topology. To characterize the distribution and reception of the global equilibrium objective by each phase, a diagonal traction matrix $B=diag(b_{1},b_{2},b_{3},b_{4})$ is introduced. Here, *b*_*i*_ = 1 indicates that phase *i* directly receives the objective information, while *b*_*i*_ = 0 means there is no direct connection. Given that all phases are capable of information exchange among themselves and also have direct access to the global equilibrium objective (i.e., *B* = *I* in this study), the topological relationship among the phases constitutes a fully connected graph, whose adjacency matrix *A*, in-degree matrix *D*, and Laplacian matrix *L* are given by:

$$A=[\begin{array}{cccc} 0 & 1 & 1 & 1\\ 1 & 0 & 1 & 1\\ 1 & 1 & 0 & 1\\ 1 & 1 & 1 & 0 \end{array}],\;D=[\begin{array}{cccc} 3 & 0 & 0 & 0\\ 0 & 3 & 0 & 0\\ 0 & 0 & 3 & 0\\ 0 & 0 & 0 & 3 \end{array}],\;L=[\begin{array}{cccc} 3 & -1 & -1 & -1\\ -1 & 3 & -1 & -1\\ -1 & -1 & 3 & -1\\ -1 & -1 & -1 & 3 \end{array}]$$ (2)

To ensure the rationality and implementability of the control objective, this paper defines the equilibrium objective l^*^(*k* + 1) as the desired average queue length across all measured phases for the subsequent control cycle. This objective is designed to effectively characterize the overall traffic equilibrium level of the intersection.

Since the queue length *l*_*i*_(*k*) across all phases *i* is fully observable at the conclusion of control cycle *k*, the most interpretable and implementable form of the equilibrium objective for cycle k+1 is calculated using this currently available information:

$$l^{*}(k+1)=\frac{1}{4}\sum_{i=1}^{4}l_{i}(k).$$ (3)

This definition ensures that the control objective is both interpretable (based on current status) and fully implementable in real-world traffic systems.

**Fig 2:**
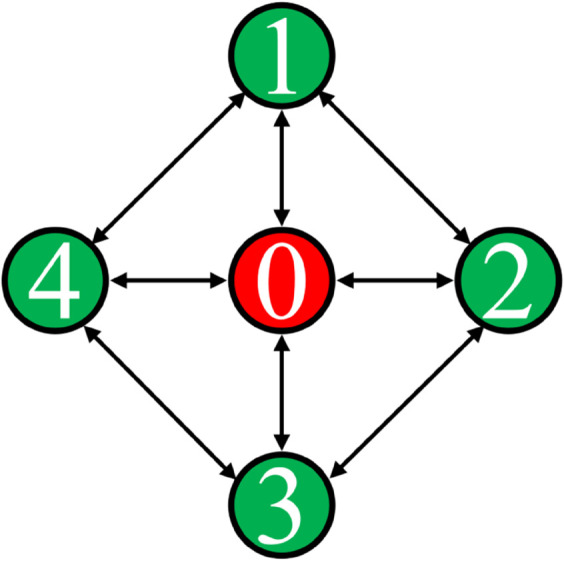
Integrated communication topology of the global equilibrium objective and signal phases.

To provide a more intuitive understanding of the allocation relationship reflected by matrix *B*, a topology diagram is introduced for visualization. As shown in Fig 2, the central node (indexed as 0) represents the global equilibrium objective l^*^(*k*), while the four surrounding nodes correspond to the four signal phases. Their communication and information exchange relationships are consistent with those defined by matrices *L* and *B*. In the diagram, arrows indicate the directed communication links between nodes. This topology organically integrates the physical phases with the global equilibrium objective and helps to clarify the overall framework and information flow characteristics of the intersection’s cooperative control structure.

Within this framework, the leader-based distributed error, *H*_*i*_(*k* + 1), is introduced to characterize the prospective deviations between each phase, its neighbors, and the equilibrium objective, formulated as follows:

$$H_{i}(k+1)=\sum_{j\in N_{i}}a_{ij}(l_{i}(k+1)-l_{j}(k+1))+b_{i}(l^{*}(k+1)-l_{i}(k+1))$$ (4)

Remark 2: The fundamental goal of the proposed control scheme is to design a control law using data available at cycle *k* that drives the future distributed error $H_{i}(k\;+\;1)$ to zero. This objective is deeply rooted in the theory of multi-agent cooperative control, particularly in leader-follower consensus problems [38]. In that context, driving the error to zero ensures that all agents’ states converge to the leader’s state and achieve consensus among themselves. Here, we adapt this framework to traffic control: ensuring $H_{i}(k\;+\;1)\to 0$ simultaneously achieves two critical traffic engineering objectives at the next time step: (1) balancing the queue lengths across all phases to prevent disparity; and (2) guiding the overall queue lengths toward the cooperative control objective, thereby maintaining traffic stability at the intersection.

### DoS attack model

In the considered isolated intersection traffic signal control scenario, the controller relies on queue length information from detectors for real-time traffic state adjustment. However, DoS attacks can disrupt the communication link between detectors and the controller, leading to the loss of queue length data at certain times. To model this, we introduce a binary variable, $\alpha_{n}(k)$, to represent the link’s status during cycle *k*. Specifically, $\alpha_{n}(k)=1$ indicates a normal link where the controller receives accurate queue length data. Conversely, $\alpha_{n}(k)=0$ signifies a successful DoS attack, resulting in a complete loss of this information.

$$\alpha_{n}\left(k\right)=\left\{ \begin{array}{cc} 1 & \text{if the queue length }l_{n}(k)\text{ is available to intersection }n\text{ at k-th cycle }\\ 0 & \text{if the queue length }l_{n}(k)\text{ is unavailable to intersection }n\text{ at k-th cycle } \end{array}\right.$$ (5)

Furthermore, $\alpha_{n}(k)$ can be modeled as an independent and identically distributed Bernoulli random variable, whose probability distribution is given by:

$$\mathbb{P}[\alpha_{n}(k)=1]=1-p,\;\mathbb{P}[\alpha_{n}(k)=0]=p,$$ (6)

where p∈[0,1] denotes the probability that the DoS attack succeeds, resulting in a complete loss of the queue length information.

In summary, this model describes the impact of DoS attacks on the traffic signal control system of an isolated intersection through a simple yet intuitive probabilistic framework. The essence lies in the following: the decision-making basis of the controller shifts from complete traffic state information to partially randomly missing observations, which lays a theoretical foundation for the subsequent resilient controller design and performance degradation analysis.

In order to improve the readability, the symbols used in this paper are listed in Table 1 below.

**Table 1:** Symbols.

Symbol	Meaning
*G*,*V*,*E*	Directed graph, set of agents, and set of edges
*A*,*a*_*ij*_	Adjacency matrix and its element representing the link from agent *j* to *i*
*D*,*d*_*i*_	In-degree matrix and the in-degree of agent *i*
*L*	Laplacian matrix of the graph *G*
*B*,*b*_*i*_	Traction matrix and its element for the link to the global objective
*N*_*i*_	The set of neighboring agents for agent *i*
*k*	Discrete time index representing the control cycle
*l*_*i*_(*k*)	Queue length of phase *i* at cycle *k*
*g*_*i*_(*k*)	Green time duration for phase *i* at cycle *k*
$\Delta g_{i}(k)$	Change in green time, *g*_*i*_(*k*)−*g*(*k*−1)
*C*	Total signal cycle length
*t*_*l*_	Lost time per cycle
l^*^(*k* + 1)	Equilibrium objective (desired average queue length) for cycle *k* + 1
*H*_*i*_(*k* + 1)	The fundamental leader-based distributed error for cycle *k* + 1
${\tilde{H}}_{i}(k)$	Weighted distributed error at cycle *k*
${\tilde{H}}_{i,d}(k)$	DoS-compensated weighted distributed error at cycle *k*
$\Delta H_{i}(k+1)$	Change in the distributed error, $H_{i}(k+1)-H_{i}(k)$
$I_{i}(\cdot ),F_{i}(\cdot )$	Unknown nonlinear functions for system and error dynamics
$\Psi_{i}(k)$	The true (unknown) pseudo partial derivative (PPD) of the system
${\hat{\Psi }}_{i}(k)$	The estimated value of the system PPD, $\Psi_{i}(k)$
$\Phi_{i}(k)$	The true (unknown) PPD of the ideal controller
${\hat{\Phi }}_{i}(k)$	The estimated value of the controller PPD, $\Phi_{i}(k)$
$\alpha_{n}(k)$	Binary variable indicating communication status (1: normal, 0: attack)
*p*	Probability of a successful DoS attack
$\varsigma_{i},\iota_{i}$	Weighting factors in the parameter estimation cost function
$\rho_{i},{\underset{―}{\rho }}_{i}$	Upper and lower reset bounds for ${\hat{\Phi }}_{i}(k)$
$z_{i},{\underset{―}{z}}_{i}$	Upper and lower reset bounds for ${\hat{\Psi }}_{i}(k)$
$g_{min},g_{max}$	Minimum and maximum green time constraints
*K*_*max*_	Total number of control cycles in the simulation
AQL, AWT	Average queue length and average waiting time

## Methods

### DMFAC framework formulation

By substituting Eq (1) and Eq (3) into Eq (4), the leader-based distributed error can be expressed in the following form:

$$H_{i}(k+1)=\sum_{j\in N_{i}}a_{ij}(I_{i}(l_{i}(k),g_{i}(k))-I_{j}(l_{j}(k),g_{j}(k)))\;\;+b_{i}\left(\frac{1}{4}\sum_{i=1}^{4}l_{i}(k)-I_{i}(l_{i}(k),g_{i}(k))\right)$$ (7)

Since $H_{i}(k\;+\;1)$ is a nonlinear function of *g*_*i*_(*k*), *g*_*j*_(*k*), *l*_*i*_(*k*), and *l*_*j*_(*k*), $H_{i}(k\;+\;1)$ can be described as the following more general form:

$$H_{i}(k+1)=F_{i}(l_{i}(k),g_{i}(k),l_{j}(k),g_{j}(k))$$ (8)

where $F_{i}(\cdot )$ is an augmented nonlinear function.

When adopting the DMFAC framework, the following fundamental assumptions must be satisfied:

Assumption 1 [20]: The partial derivative of $I_{i}(\cdot )$ with respect to *g*_*i*_(*k*) is continuous.

Assumption 2 [20]: The nonlinear function $I_{i}(\cdot )$ is generalized Lipschitz, i.e.,

$$\left|\Delta l_{i}(k+1)|\leq \ell_{i}|\Delta g_{i}(k)\right|$$ (9)

where $\Delta g_{i}(k)=g_{i}(k)-g_{i}(k-1)$, for $|\Delta g_{i}(k)|\neq 0$, $\Delta l_{i}(k+1)=l_{i}(k+1)-l_{i}(k)$ denotes the increment of the queue length of the *i*-th phase in the k+1-th cycle, and $\ell_{i}>0$ is a positive constant.

Assumption 3 [38]: The input increment between the green time of the *i*-th phase and the green time of the *j*-th phase satisfies the numerical relationship:

$$\left|\Delta g_{j}(k)|\leq o_{i}|\Delta g_{i}(k)\right|$$ (10)

where *o*_*i*_>0 is a bounded positive constant.

Remark 3: Assumptions 1–3 constitute the fundamental assumptions of the DMFAC design framework. From an engineering practice viewpoint, all these assumptions are reasonable and acceptable. Specifically, Assumption 1 corresponds to the conventional constraints for general nonlinear systems; Assumption 2 specifies the upper bound of the system output change rate driven by variations in control inputs—for instance, in traffic systems, the change in queue length is constrained by the finite variation of green time; Assumption 3 reflects the system’s energy constraint, which can be naturally satisfied as long as the green time of each phase is bounded. Moreover, a common prerequisite in the DMFAC framework is that the communication topology contains a directed spanning tree. For the single-intersection traffic signal control system investigated in this paper, its communication topology inherently satisfies this property. Thus, this paper does not list it as a separate assumption, but this condition is presumed to hold in subsequent theoretical analyses.

Lemma 1 [20]: If Eq (1) satisfies Assumptions 1–3 for all *i* and *k*, then it can be equivalently expressed as the CFDL data model, which is given by:

$$\Delta H_{i}(k+1)=\Psi_{i}(k)\Delta g_{i}(k)$$ (11)

where $\Psi_{i}(k)$ denotes the PPD and satisfies $|\Psi_{i}(k)|\leq \varpi_{i}$, where $\varpi_{i}$ is a constant related to $\ell_{i}$ and *o*_*i*_.

### Controller dynamic linearization

The objective of this paper is to design a purely data-driven CDL-DMFAC control scheme based on the data model in Eq (11), such that the control system satisfies the equilibrium objective in Eq (3). Theoretically, if there exists an ideal controller that can achieve the control objective, it can provide a benchmark for the design of the control law. Existing studies have designed various control systems based on the ideal controller within the MFAC framework to solve the regulation problem of nonlinear systems. Such methods are referred to as direct MFAC, distinguishing them from the prototype indirect MFAC.

Based on the above discussion, it is assumed that there exists an ideal distributed controller of the following form:

$$g_{i}(k)=G_{i}(g_{i}(k-1),H_{i}(k+1))$$ (12)

Assumption 4 [33]: The partial derivative of $G_{i}(\cdot )$ with respect to the distributed error *H*_*i*_(*k* + 1) is continuous.

Assumption 5 [33]: $G_{i}(\cdot )$ satisfies the generalized Lipschitz condition, i.e.,

$$\left|\Delta g_{i}(k)|\leq \sigma_{i}|\Delta H_{i}(k+1)\right|$$ (13)

Remark 4: Assumption 4 belongs to the classical prior conditions for ideal controller design. Assumption 5 indicates that bounded distributed error increments produce bounded control signals; in other words, the controller should be an energy-consuming unit.

Lemma 2 [33]: If Assumptions 4, 5, and $|\Delta g_{i}(k)|\neq 0$ are all satisfied, then the equivalent CFDL model of the ideal controller is:

$$\Delta g_{i}(k)=\Phi_{i}(k)\Delta H_{i}(k+1)$$ (14)

Note that there exists a non-causal term in Eq (14), so this form cannot be directly used for real-time signal control. However, as an ideal controller, its output can achieve perfect one-step-ahead tracking, i.e., $H_{i}(k\;+\;1)=0$. Based on this property, Eq (14) can be transformed into an implementable control law form:

$$\Delta g_{i}(k)=-\Phi_{i}(k)H_{i}(k)$$ (15)

Remark 5: Eq (15) represents the structure of the designed controller. The significance of this process is that the control system design problem can be replaced by a controller parameter identification problem, and the relationship between the control input and distributed coordination error can be obtained through the data model Eq (11). In the next section, we will design a signal control scheme based on the constructed data model Eq (11) and Eq (15). It should be noted that both the PPD in Eq (11) and the PPD in Eq (15) are unknown. Therefore, it is necessary to estimate them using the I/O data of the traffic signal control system.

### CDL-DMFAC algorithm design

To enhance the coordination capability of distributed errors, a dynamic weighting mechanism is introduced, and the weighted distributed error is defined as:

$${\tilde{H}}_{i}(k)=W_{i}(k)H_{i}(k)$$ (16)

where ${\tilde{H}}_{i}(k)$ is the weighted distributed error, and the dynamic weight factor $W_{i}(k)=l_{i}(k)/\sum_{i=1}^{4}l_{i}(k)$.

Remark 6: In traffic signal coordination control, the distributed error *H*_*i*_(*k*) is usually used to characterize the queue balance deviation between phases. However, in practical environments, the queue lengths of different phases vary significantly. If the original error is directly used, the controller misjudges the control urgency of these imbalances. It underestimates the severity of deviations in heavily congested phases, treating a critical risk of queue spillback with the same priority as a minor fluctuation in light traffic. For this reason, in the subsequent algorithm design, all distributed errors are replaced by the weighted distributed error ${\tilde{H}}_{i}(k)$.

First, to capture the dynamic characteristics of the controlled system, we estimate the system PPD $\Psi_{i}(k)$ defined in Eq (11). The following estimation criterion is proposed:

$$J(\Psi_{i}(k))=\frac{1}{2}{\left(\Delta {\tilde{H}}_{i}(k)-\Psi_{i}(k)\Delta g_{i}(k-1)\right)}^{2}+\frac{1}{2}\iota_{i}{\left(\Psi_{i}(k)-{\hat{\Psi }}_{i}(k-1)\right)}^{2}$$ (17)

where $\iota_{i}>0$ is a weight factor.

Minimizing Eq (17) with respect to $\Psi_{i}(k)$ yields the estimation algorithm for the system PPD:

$${\hat{\Psi }}_{i}(k)={\hat{\Psi }}_{i}(k-1)+\frac{\Delta g_{i}(k-1)\Delta {\tilde{H}}_{i}(k)}{\iota_{i}+{\left|\Delta g_{i}(k-1)\right|}^{2}}-\frac{{\hat{\Psi }}_{i}(k-1)\Delta g_{i}^{2}(k-1)}{\iota_{i}+{\left|\Delta g_{i}(k-1)\right|}^{2}}$$ (18)

where ${\hat{\Psi }}_{i}(k)$ is the estimated value of $\Psi_{i}(k)$. Similar to the projection algorithm constraints, a small positive constant ${\underset{―}{z}}_{i}\in (0,z_{i})$ is defined, and the corresponding reset mechanism is given by:

$${\hat{\Psi }}_{i}(k)={\hat{\Psi }}_{i}(1),\text{if }{\hat{\Psi }}_{i}(k)<{\underset{―}{z}}_{i}\text{ or }{\hat{\Psi }}_{i}(k)>z_{i}$$ (19)

With the estimated system parameter ${\hat{\Psi }}_{i}(k)$ obtained, we proceed to estimate the controller parameter $\Phi_{i}(k)$ under the framework of the controller data model in Eq (15). The estimation criterion is expressed as:

$$J(\Phi_{i}(k))=\frac{1}{2}{\left({\tilde{H}}_{i}(k+1)\right)}^{2}+\frac{1}{2}\varsigma_{i}{\left(\Phi_{i}(k)-{\hat{\Phi }}_{i}(k-1)\right)}^{2}$$ (20)

where $\varsigma_{i}>0$ is a weight factor.

Substituting the models in Eqs (11) and 15 into the criterion in Eq (20) and using $\partial J/\partial \Phi_{i}(k)=0$, we obtain the controller parameter update law:

$${\hat{\Phi }}_{i}(k)={\hat{\Phi }}_{i}(k-1)+\frac{{\tilde{H}}_{i}^{2}(k){\hat{\Psi }}_{i}(k)}{\varsigma_{i}+{\left|{\hat{\Psi }}_{i}(k){\tilde{H}}_{i}(k)\right|}^{2}}-\frac{{\hat{\Phi }}_{i}(k-1){\left|{\hat{\Psi }}_{i}(k){\tilde{H}}_{i}(k)\right|}^{2}}{\varsigma_{i}+{\left|{\hat{\Psi }}_{i}(k){\tilde{H}}_{i}(k)\right|}^{2}}$$ (21)

where ${\hat{\Phi }}_{i}(k)$ is the estimated value of $\Phi_{i}(k)$. Note that in Eq (21), the unknown true system PPD $\Psi_{i}(k)$ has been replaced by its estimated value ${\hat{\Psi }}_{i}(k)$, which is calculated via Eq (18) derived above.

To better characterize the time-varying characteristic of ${\hat{\Phi }}_{i}(k)$, the following reset condition is introduced:

$${\hat{\Phi }}_{i}(k)={\hat{\Phi }}_{i}(1),\;\text{if}\;{\hat{\Phi }}_{i}(k)<-\rho_{i}\;\text{or}\;{\hat{\Phi }}_{i}(k)>-{\underset{―}{\rho }}_{i}$$ (22)

where $\rho_{i}>{\underset{―}{\rho }}_{i}>0$.

Remark 7: It can be seen from Eq (22) that the sign of ${\hat{\Phi }}_{i}(k)$ is constrained to maintain consistency with the initial negative feedback characteristic, which is supported by general control theories. Meanwhile, since the controller structure is artificially designed, the value range of its parameters can be preset in the design stage.

To summarize, the proposed controller can be finally expressed as:

$$\Delta g_{i}(k)=-{\hat{\Phi }}_{i}(k){\tilde{H}}_{i}(k)$$ (23)

### Green time constraint handling

In addition to the aforementioned error constraints related to controller design, in practical traffic signal control, it is also necessary to consider the physical feasibility constraints of green light duration and signal cycle.

To avoid excessively long or short green light time for a single phase, it is necessary to set upper and lower limit constraints on the green light time of each phase, i.e.:

$$g_{i}(k)=\left\{ \begin{array}{cc} \min\{g_{i}(k),g_{max}\} & g_{i}(k)>g_{max}\\ g_{i}(k) & g_{min}\leq g_{i}(k)\leq g_{max}\\ \max\{g_{i}(k),g_{min}\} & g_{i}(k)<g_{min} \end{array}\right.$$ (24)

where *g*_*min*_ and *g*_*max*_ are the preset minimum and maximum values, respectively.

On this basis, the signal cycle *C* of the intersection needs to satisfy the overall constraint conditions. The signal cycle refers to the complete duration from the start of a certain phase to entering that phase again, which is composed of the green light time and yellow light time of each phase. Therefore, the cycle constraint can be expressed as:

$$C(k)=\sum_{i=1}^{4}g_{i}(k)+t_{l}$$ (25)

where *t*_*l*_ represents the total lost time per cycle. Specifically, *t*_*l*_ is defined as the cumulative sum of the intergreen intervals (configured as yellow light time) for all phase transitions. In this study, we set the yellow interval to 3s per phase. This ensures that vehicles from the previous phase have sufficient clearance time before the next phase begins, thereby physically preventing inter-phase conflicts.

Through the above constraint conditions, the balance between safety and efficiency in signal timing can be ensured, thereby improving the rationality and effectiveness of traffic control.

### Resilient CDL-DMFAC under DoS attacks

To mitigate the impact of DoS attacks, a compensation mechanism is developed, drawing inspiration from the method proposed in [37]:

$${\tilde{H}}_{i,d}(k)=\alpha_{n}(k){\tilde{H}}_{i}(k)+\left(1-\alpha_{n}(k)\right){\tilde{H}}_{i}(k-1)$$ (26)

Remark 8: It can be observed that the attack compensation mechanism consists of two components, namely ${\tilde{H}}_{i}(k)$ and ${\tilde{H}}_{i}(k-1)$. If the DoS attack in the transmission network is successful, then $\alpha_{n}(k)=0$ and ${\tilde{H}}_{i,d}(k)={\tilde{H}}_{i}(k-1)$, which means the controller will use the latest received data packet ${\tilde{H}}_{i}(k-1)$ stored in the buffer to compensate for the impact of the DoS attack.

**Fig 3:**
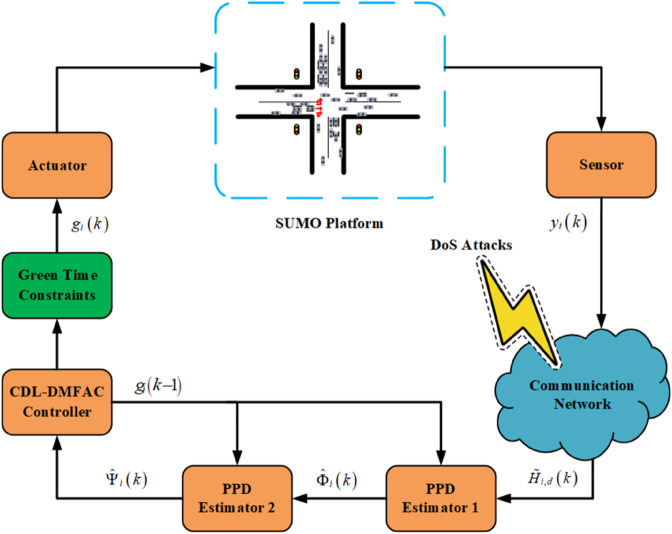
Procedure of the resilient CDL-DMFAC Algorithm against DoS attacks.

By combining Eqs (17)–(23) and incorporating the compensation mechanism in Eq (26) against DoS attacks, a resilient DMFAC algorithm (see Eqs (27)–(29)) is developed, and its detailed procedure is illustrated in Fig 3 and Algorithm 1. It should be noted that the reset mechanisms corresponding to Eqs (19) and (22) remain unchanged under DoS conditions and are therefore not elaborated here.

$${\hat{\Psi }}_{i}(k)={\hat{\Psi }}_{i}(k-1)+\frac{\Delta g_{i}(k-1)\Delta {\tilde{H}}_{i,d}(k)}{\iota_{i}+{\left|\Delta g_{i}(k-1)\right|}^{2}}-\frac{{\hat{\Psi }}_{i}(k-1)\Delta g_{i}^{2}(k-1)}{\iota_{i}+{\left|\Delta g_{i}(k-1)\right|}^{2}}$$ (27)

$${\hat{\Phi }}_{i}(k)={\hat{\Phi }}_{i}(k-1)+\frac{{\tilde{H}}_{i,d}^{2}(k){\hat{\Psi }}_{i}(k)}{\varsigma_{i}+{\left|{\hat{\Psi }}_{i}(k){\tilde{H}}_{i,d}(k)\right|}^{2}}-\frac{{\hat{\Phi }}_{i}(k-1){\left|{\hat{\Psi }}_{i}(k){\tilde{H}}_{i,d}(k)\right|}^{2}}{\varsigma_{i}+{\left|{\hat{\Psi }}_{i}(k){\tilde{H}}_{i,d}(k)\right|}^{2}}$$ (28)

$$\Delta g_{i}(k)=-{\hat{\Phi }}_{i}(k){\tilde{H}}_{i,d}(k)$$ (29)

Remark 9: It can be seen from the proposed control strategy in Eqs (27)–(29) that when the data transmission channel is subjected to DoS attacks, rendering the controller unable to obtain the latest queuing status, the compensation mechanism will be automatically triggered. It maintains the update of the control law by using the available information from the previous time step, thus ensuring the implementability of the algorithm and the continuity of the control input. It should be emphasized that the method in this paper does not rely on accurate model parameters of the intersection traffic system during the design process, but constructs the distributed control law purely based on I/O data. This is different from traditional distributed control methods that rely on traffic flow dynamics models. The data-driven design enables the proposed control framework to better adapt to the complex and changeable intersection environment in urban traffic, exhibiting stronger robustness and engineering application value in scenarios with uncertainties, random disturbances, and difficulties in obtaining accurate models.

**Algorithm 1 Design of the resilient CDL-DMFAC strategy under DoS attacks.**

**Initialization:**

1: Given controller parameters $\varsigma_{i},\iota_{i}$; PPD reset bounds $\rho_{i},{\underset{―}{\rho }}_{i},z_{i},{\underset{―}{z}}_{i}$; green time constraints $g_{min},g_{max}$; cycle length *C*; and simulation duration to obtain *K*_*max*_.

2: For each phase i∈{1,2,3,4}, set initial values: green time *g*_*i*_(1), queue length *l*_*i*_(1), controller PPD ${\hat{\Phi }}_{i}(1)$, and system PPD ${\hat{\Psi }}_{i}(1)$.

3: Set control cycle k←2.

4: **for** *k* = 2 to *K*_*max*_ **do** **State Evaluation & Error Calculation:**

5:   Collect current queue length *l*_*i*_(*k*) for all phases *i*.

6:   Compute the equilibrium objective l^*^(*k*) via Eq (3).

7:   Compute the weighted distributed error ${\tilde{H}}_{i}(k)$ via Eqs (4) and (16).

**Resilience & Compensation:**

8:   Obtain communication status $\alpha_{n}(k)$.

9:   **if** communication is normal ($\alpha_{n}(k)=1$) **then**

10:    ${\tilde{H}}_{i,d}(k)\leftarrow {\tilde{H}}_{i}(k)$.

11:   **else** ▷ Under DoS attack ($\alpha_{n}(k)=0$)

12:    ${\tilde{H}}_{i,d}(k)\leftarrow {\tilde{H}}_{i}(k-1)$. ▷ Activate compensation mechanism

13:   **end if**

**Parameter Estimation & Control Update:**

14:   Update system PPD ${\hat{\Psi }}_{i}(k)$ online via Eq (27) and apply reset logic in Eq (19).

15:   Update controller PPD ${\hat{\Phi }}_{i}(k)$ online via Eq (28) and apply reset logic in Eq (22).

16:   Compute control law via Eq (29) to obtain green time increment $\Delta g_{i}(k)$.

17:   Update green time: $g_{i}(k)\leftarrow g_{i}(k-1)+\Delta g_{i}(k)$.

**Constraint Application & Actuation:**

18:   Apply boundary constraints to ensure $g_{i}(k)\in [g_{min},g_{max}]$.

19:   Normalize green times for all phases to satisfy the total cycle length *C*.

20:   Apply the final green time plan $\left\{g_{1}(k),...,g_{4}(k)\right\}$ to the traffic signal.

21:   k←k+1;.

22: **end for**

## Experiment

### Experimental setup

**Fig 4:**
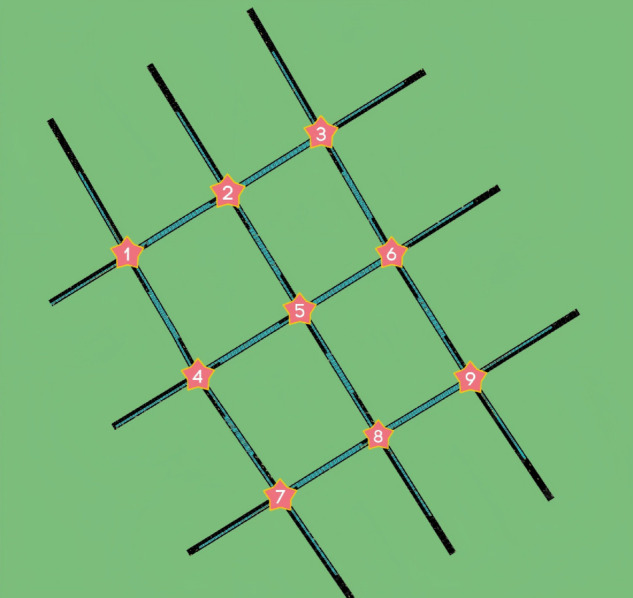
Road network representations of the study area.

The experiment was conducted on a real-world road network in Tongzhou District, Beijing, comprising nine four-phase signalized intersections. The network was modeled in the Simulation of Urban Mobility (SUMO) platform using road network topology data extracted from OpenStreetMap (OSM), as shown in Fig 4. We confirm that the collection and use of this map data complied with the terms and conditions of the OpenStreetMap Foundation, specifically adhering to the Open Database License (ODbL) v1.0. Furthermore, the traffic flow data used in the experiments were synthetically generated within the simulation platform based on a Poisson distribution model. Finally, the proposed CDL-DMFAC algorithm was integrated via the Traffic Control Interface (TraCI) for real-time, closed-loop control.

To evaluate the algorithm’s performance and robustness, experiments were designed across three traffic demand levels and three DoS attack scenarios. Vehicle arrivals followed a Poisson distribution with demand set to low (400 veh/h/lane), medium (800 veh/h/lane), and high (1600 veh/h/lane) to represent sparse, moderate, and near-saturation conditions, respectively. The attack scenarios included: (1) no DoS attack (baseline); (2) single-point DoS attack on one randomly selected intersection; and (3) multi-point DoS attacks on five randomly selected intersections.

Each simulation run lasted for 3600 s. To ensure a fair comparison, all tested control methods operated under the same global settings: a fixed cycle length of *C* = 132 s, green times constrained between $g_{min}=15$ s and $g_{max}=60$ s, and a uniform yellow time of 3 s. The specific parameters for the proposed CDL-DMFAC controller are detailed in Table 2 and were applied consistently across all scenarios. To ensure statistical validity, results were averaged over multiple runs with different random seeds.

**Table 2:** Key parameters of the CDL-DMFAC-based adaptive traffic signal control algorithm.

Symbol	Name	Default Value
${\hat{\Psi }}_{i}(1)$	Initial pseudo-partial derivative	0.5
${\hat{\Phi }}_{i}(1)$	Initial Control gain	–0.5
*g*_*i*_(1)	Initial green time	[20, 40, 20, 40]
*y*_*i*_(1)	Initial queue length	0
$\varsigma_{i}$	Forgetting factor	1.0
$\iota_{i}$	Forgetting factor	1.0
$\underset{―}{\rho_{i}}$	${\hat{\Phi }}_{i}(k)$ reset lower bound	$1\times 10^{-5}$
$\rho_{i}$	${\hat{\Phi }}_{i}(k)$ reset upper bound	0.3
${\underset{―}{z}}_{i}$	${\hat{\Psi }}_{i}(k)$ reset lower bound	$1\times 10^{-5}$
*z*_*i*_	${\hat{\Psi }}_{i}(k)$ reset upper bound	0.3
*L*	Laplacian matrix	see Eq (2)
*B*	Traction matrix	*I*

### Comparison with baseline methods

### Evaluation metrics

To validate the overall advantages of the proposed CDL-DMFAC method under varying traffic demand levels and DoS attack scenarios, two fundamental indicators for traffic signal control performance are selected: the average queue length (AQL) and the average waiting time (AWT). AQL is a measure of spatial congestion and capacity utilization, quantified by the average number of vehicles queued over the evaluation period. AWT is a measure of temporal delay and driver experience, quantified by the average waiting time per vehicle to clear the intersection.

The calculation formulas for AQL and AWT, which serve as the comprehensive performance metrics, are defined as follows:

$$\text{AQL}=\frac{1}{K_{max}}\sum_{k=1}^{K_{max}}\left(\frac{1}{N_{\text{phase}}}\sum_{i=1}^{N_{\text{phase}}}l_{i}(k)\right)$$ (30)

$$\text{AWT}=\frac{1}{K_{max}}\sum_{k=1}^{K_{max}}\left(\frac{1}{N_{\text{veh}}}\sum_{j=1}^{N_{\text{veh}}}W_{j}\right)$$ (31)

where *K*_*max*_ is the total number of control cycles; $N_{\text{phase}}$ is the total number of phases (or measured approaches) in the intersection; $N_{\text{veh}}$ is the total number of vehicles that have passed through the intersection during the evaluation period; and *W*_*j*_ is the waiting time of vehicle *j*.

To ensure the comprehensiveness and representativeness of the comparison, four typical baseline methods are selected, covering conventional fixed-time control strategies and data-driven adaptive control approaches, thereby reflecting the improvements of CDL-DMFAC from multiple perspectives in terms of traffic efficiency and attack resilience. The details are as follows:

1. **Fixed-time control (FT)** [1] — A classical static signal control strategy in which the cycle length and green-time split for each phase are pre-determined based on historical traffic data prior to system deployment and remain unchanged during operation. This method is simple to implement and highly stable, but it cannot adapt to real-time traffic flow fluctuations and is more suitable for scenarios with relatively stable demand.
2. **Proportional-integral-derivative control (PID)** [4] — A classical closed-loop feedback control algorithm that dynamically adjusts the green time of the next cycle based on the deviation between the measured and target queue length (or average delay), using proportional, integral, and derivative terms. Although PID control offers fast response, its adaptability is limited in highly nonlinear and time-varying traffic environments.
3. **Queue length feedback control (QLFB)** [5] — A feedback-based signal control method that uses real-time queue length information. The system continuously monitors the queue length at each approach and compares it with a target value, adjusting the green time of the corresponding phase proportionally to the deviation. This method is simple to implement and relatively robust, but its control logic is relatively simplistic, which may lead to oscillations or delayed responses in complex scenarios.
4. **Compact form dynamic linearization (CFDL)** [20] — A data-driven control method for nonlinear dynamic systems that does not rely on an exact mathematical model of the system. Instead, it constructs a compact form dynamic linearization model using only the system’s input–output data. This method enables online parameter updating and adaptive control, allowing it to accommodate dynamic variations in traffic flow to a certain extent.

## Results analysis

### Scenario 1: No DoS attack

**Fig 5:**
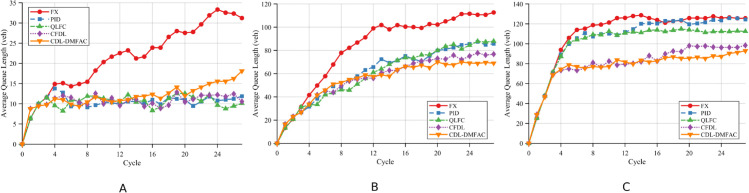
Comparison of the proposed method and baseline approaches in average queue length at intersections under Scenario 1 across different traffic demand levels. A: Low demand. B: Medium demand. C: High demand.

Scenario 1 serves as the baseline case, where communication and control at all intersections function normally without any DoS attacks. The corresponding performance results are illustrated in Fig 5 and summarized in Tables 3 and 4.

**Table 3:** Comparison of average queue lengths (vehicles) under different traffic demands under Scenario 1.

	FT	PID	QLFB	CFDL	CDL-DMFAC	Improvement
Low	21.43	10.18	10.14	10.44	11.74	45.2%
Middle	81.98	57.76	59.19	55.85	54.07	34.0%
High	109.52	104.54	99.48	79.42	76.36	30.3%

Table notes: “Improvement” is the percentage reduction in the average queue length (AQL) of CDL-DMFAC relative to fixed-time (FT) control under the same traffic conditions.

**Table 4:** Comparison of average waiting times (s/veh) under different traffic demands under Scenario 1.

	FT	PID	QLFB	CFDL	CDL-DMFAC	Improvement
Low	115.94	98.71	86.36	97.71	103.89	10.4%
Middle	440.67	304.44	293.96	354.72	344.53	21.8%
High	771.61	648.24	538.89	539.4	515.87	33.1%

Table notes: “Improvement” is the percentage reduction in the average waiting time (AWT) of CDL-DMFAC relative to fixed-time (FT) control under the same traffic conditions.

Under normal operating conditions without DoS attacks, the differences in AQL and AWT among all methods are minimal in the low-traffic scenario, indicating that traffic demand at this level has not yet exerted substantial pressure on the network. As traffic volume increases from medium to high, the performance of traditional control methods declines noticeably, whereas the proposed CDL-DMFAC method consistently maintains lower queue lengths and vehicle delays. Notably, in the high-traffic scenario, the CDL-DMFAC method achieves significantly lower AQL and AWT than the other methods, with reductions of approximately 30.3% and 33.1%, respectively, demonstrating that its distributed cooperative optimization strategy can more effectively mitigate congestion and reduce vehicle delays.

**Fig 6:**
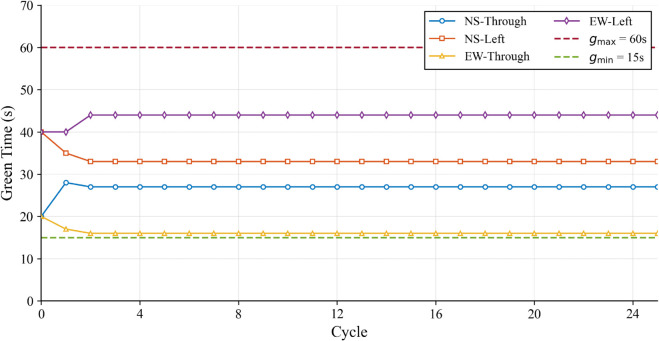
Control input (green signal duration) trajectories of Intersection 5 under high traffic demand in Scenario 1.

Furthermore, to verify the physical feasibility of the proposed strategy, the dynamic variations of the control input were analyzed. Intersection 5 was selected as the representative subject due to its critical role in the subsequent DoS attack scenarios, and the high traffic demand condition was chosen to validate the constraint handling mechanism under the most challenging operational stress. The green signal duration trajectories are depicted in Fig 6. As shown, the controller dynamically adjusts the signal timings in response to traffic fluctuations. Crucially, the control input strictly adheres to the preset safety boundaries (indicated by dashed lines at *g*_*min*_ = 15s and *g*_*max*_ = 60s), ensuring that the generated signal timings are physically executable.

### Scenario 2: Single-point DoS attack

**Fig 7:**
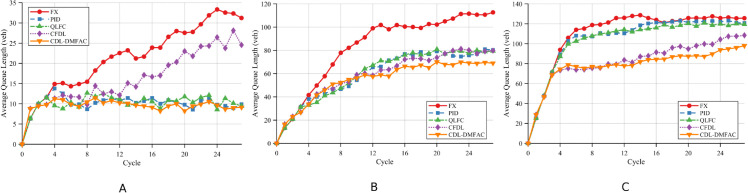
Comparison of the proposed method and baseline approaches in average queue length at intersections under Scenario 2 across different traffic demand levels. A: Low demand. B: Medium demand. C: High demand.

In Scenario 2, the target node of the single-point DoS attack is Intersection 5, with the attack success probability *p* set to 50%. Under this setting, Fig 7, together with Tables 5 and 6, presents the performance of each control strategy in Scenario 2 in terms of AQL and AWT under different traffic demand levels.

**Table 5:** Comparison of average queue lengths (vehicles) under different traffic demands under Scenario 2.

	FT	PID	QLFB	CFDL	CDL-DMFAC	Improvement
Low	21.43	9.97	10.09	16.17	9.4	56.14%
Middle	81.98	58.79	59.31	58.17	54.07	34.04%
High	109.52	104.17	102.21	81.62	77.14	29.57%

Table notes: “Improvement” is the percentage reduction in the average queue length (AQL) of CDL-DMFAC relative to fixed-time (FT) control under the same traffic conditions.

**Table 6:** Comparison of average waiting times (s/veh) under different traffic demands under Scenario 2.

	FT	PID	QLFB	CFDL	CDL-DMFAC	Improvement
Low	115.94	96.54	89.51	140.78	93.95	18.97%
Middle	440.67	295.39	292.13	360.69	344.53	21.82%
High	771.61	676.88	544.45	525.01	491.57	36.29%

Table notes: “Improvement” is the percentage reduction in the average waiting time (AWT) of CDL-DMFAC relative to fixed-time (FT) control under the same traffic conditions.

Under low-traffic conditions, the impact of the single-point DoS attack is primarily confined to the targeted intersection and its neighboring nodes, exerting minimal disruption on the overall network operation. Benefiting from its DoS compensation mechanism, the CDL-DMFAC method is able to maintain an advantageous signal timing plan even under partial information loss, reducing AQL and AWT by 56.14% and 18.97%, respectively, compared with the FT method, thereby demonstrating a certain degree of local disturbance-resistance.

When traffic demand rises to the medium level, the queue buildup at the attacked node begins to spread to surrounding areas. Traditional methods, lacking timely adjustments in the absence of real-time information, allow congestion to propagate more rapidly. By contrast, CDL-DMFAC employs distributed cooperative optimization to promptly adjust signal timings and allocate spatiotemporal resources effectively, thereby suppressing congestion spread. Compared with the FT method, it achieves reductions of 34.04% in AQL and 21.82% in AWT, yielding a marked improvement.

Under high-traffic conditions, the network approaches saturation, and the local disruptions caused by the single-point attack are amplified. Although the performance of all methods declines, CDL-DMFAC still maintains superior results, reducing AQL and AWT by 29.57% and 36.29%, respectively, compared with the FT method. This reflects its robustness and anti-interference capability under high-stress operating conditions, effectively mitigating the adverse global traffic efficiency impacts induced by local attacks.

**Fig 8:**
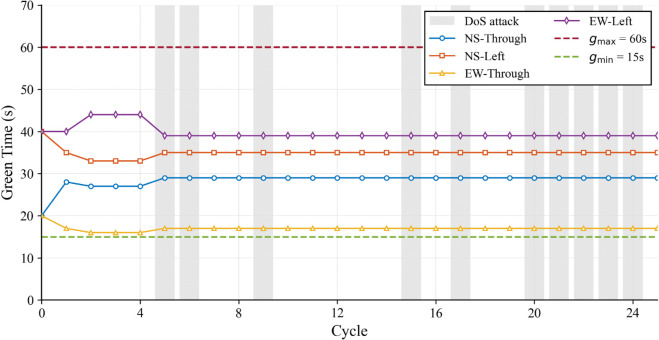
Control input (green signal duration) trajectories of Intersection 5 under high traffic demand in Scenario 2.

The operational stability underlying this superior performance is further elucidated by the control input dynamics shown in Fig 8. Here, the green signal duration for the targeted Intersection 5 is analyzed under high traffic demand. Crucially, the gray shaded region visualizes the specific duration of the active DoS attack. It can be observed that even during this period of data loss, the controller does not exhibit erratic behavior. Instead, the green time continues to fluctuate strictly within the safety boundaries ([15s,60s]). This stable control output ensures that the intersection continues to operate efficiently despite the attack, thereby preventing the local disturbance from escalating into network-wide congestion.

### Scenario 3: Multi-point DoS attacks

**Fig 9:**
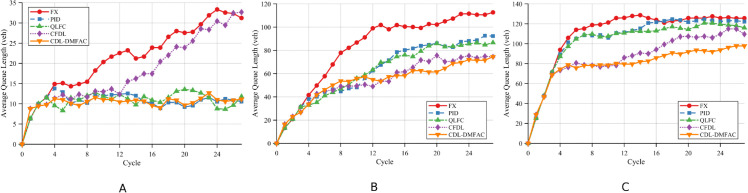
Comparison of the proposed method and baseline approaches in average queue length at intersections under Scenario 3 across different traffic demand levels. A: Low demand. B: Medium demand. C: High demand.

In Scenario 3, five critical intersections in the road network (Nodes 2, 4, 5, 6, and 8) are simultaneously subjected to DoS attacks, with the attack probability identical to that in Scenario 2. Compared with the single-point attack, the multi-point attack creates a substantially larger perception blind zone in the network, thereby leading to a significantly faster and wider spread of congestion and imposing higher robustness requirements on the traffic control system. Furthermore, Fig 9, Tables 7 and 8 present the AQL and AWT of different control strategies under various traffic demand levels.

**Table 7:** Comparison of average queue lengths (vehicles) under different traffic demands under Scenario 3.

	FT	PID	QLFB	CFDL	CDL-DMFAC	Improvement
Low	21.43	10.48	10.6	17.98	10.17	52.5%
Middle	81.98	62.24	60.83	53.8	52.84	35.5%
High	109.52	104.29	101.13	85.68	78.58	28.3%

Table notes: “Improvement” is the percentage reduction in the average queue length (AQL) of CDL-DMFAC relative to fixed-time (FT) control under the same traffic conditions.

**Table 8:** Comparison of average waiting times (s/veh) under different traffic demands under Scenario 3.

	FT	PID	QLFB	CFDL	CDL-DMFAC	Improvement
Low	115.94	97.95	92.60	149.89	101.6	12.37%
Middle	440.67	313.22	292.93	323.03	325.38	26.16%
High	771.61	699.94	586.89	628.71	491.34	36.32%

Table notes: “Improvement” is the percentage reduction in the average waiting time (AWT) of CDL-DMFAC relative to fixed-time (FT) control under the same traffic conditions.

Under low-traffic conditions, although the multi-point DoS attack results in a larger perception blind zone, the overall traffic pressure remains low, and its impact on network performance is relatively limited. Traditional methods tend to exhibit slight signal timing imbalances when partial node information is missing, whereas CDL-DMFAC, relying on an elastic DoS compensation mechanism, promptly adjusts the timing plans of the affected nodes, thus preventing the continuous accumulation of local queues. Compared with the FT method, the AQL and AWT are reduced by 52.5% and 12.37%, respectively, demonstrating stable control capability even under multi-point attacks.

When the traffic volume rises to a medium level, the large-scale state loss caused by multi-point attacks significantly accelerates congestion propagation throughout the network. Due to delayed response, traditional methods fail to effectively suppress the spread of congestion in a timely manner. CDL-DMFAC reconstructs missing information from historical data and performs distributed cooperative signal optimization, thereby delaying the expansion of local congestion to the entire network. Compared with the FT method, the AQL and AWT are reduced by 35.5% and 26.16%, respectively, indicating that the proposed method still maintains a clear advantage under medium traffic load.

Under high-traffic conditions approaching saturation, the impact of multi-point attacks on the road network is further amplified, often leading to a sharp decline in overall operational efficiency. Although the performance of all methods degrades, CDL-DMFAC maintains the continuity and balance of traffic flow guidance, mitigating the negative influence of attacked nodes on the network as a whole. Compared with the FT method, the AQL and AWT are reduced by 28.3% and 36.32%, respectively, demonstrating robustness and global optimization capability under stringent operating conditions.

**Fig 10:**
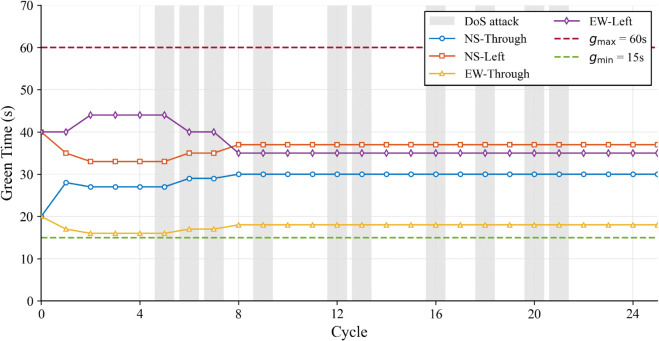
Control input (green signal duration) trajectories of Intersection 5 under high traffic demand in Scenario 3.

Finally, the stability of the control strategy under multi-point DoS attacks is verified in Fig 10. Taking Intersection 5 as an example, we analyzed its green signal duration during the attack. As shown in the figure, the gray shaded region marks the time when Intersection 5 was under attack. It can be clearly seen that even during this period, the green signal duration did not fluctuate violently or exceed the limits. Instead, it remained strictly within the safety boundaries ([15s,60s]). This proves that even when multiple intersections are attacked, the proposed method can still generate feasible and safe control signals for each local intersection.

## Conclusion

This study proposes a resilient distributed cooperative control method (CDL-DMFAC) to address intersection traffic signal control under DoS attacks. By modeling the four signal phases as independent agents and using an improved dynamic linearization strategy, the method achieves decentralized coordination and compensates for attack-induced data loss by leveraging the most recently available da ta. This model-free framework demonstrates strong distributed scalability and robustness, effectively suppressing queue growth and delay accumulation. Experimental results show that under the most challenging scenario—high traffic demand with multi-point DoS attacks—CDL-DMFAC reduces average queue length by 28.3% and average waiting time by 36.32% compared to conventional methods. These findings highlight the method’s remarkable resilience, operational stability, and promising scalability for larger transportation networks.

Future research will focus on two main directions: (1) extending the current phase-level distributed strategy to a cross-intersection cooperative control architecture, specifically incorporating objectives for downstream platoon progression and network-wide coordination to further enhance traffic efficiency; and (2) considering multiple types and hybrid forms of cyberattacks (e.g., data manipulation, false data injection) to develop defense and control frameworks that more closely reflect complex real-world traffic environments. These efforts aim to establish a solid methodological and theoretical foundation for building highly resilient intelligent transportation systems capable of withstanding cyber-physical threats.

## Supporting information

S1 RAR Intersection network, configuration and detector files. (ZIP)

S2 RAR Route configuration files for different traffic demands. (ZIP)

S3 RAR Data corresponding to the values of the figures and indicators. (ZIP)
